# The Effects of Physical Activity on the Epiphyseal Growth Plates: A Review of the Literature on Normal Physiology and Clinical Implications

**DOI:** 10.4021/jocmr477w

**Published:** 2011-02-12

**Authors:** Timothy A. Mirtz, Judy P. Chandler, Christina M. Eyers

**Affiliations:** a Division of Health Physical Education and Recreation, University of South Dakota, Vermillion, South Dakota, USA; b Department of Physical Education and Sport, Central Michigan University, Mt. Pleasant, Michigan, USA

## Abstract

**Background:**

Children need physical activity and generally do this through the aspect of play. Active play in the form of organized sports can appear to be a concern for parents. Clinicians should have a general physiological background on the effects of exercise on developing epiphyseal growth plates of bone. The purpose of this review is to present an overview of the effects of physical activity on the developing epiphyseal growth plates of children.

**Methods:**

A National Library of Medicine (Pubmed) search was initiated using the keywords and combinations of keywords "growth plate", "epiphyseal plate", "child", "exercise", and "physical activity."

**Discussion:**

Bone is a dynamic tissue with a balance of osteoblast and osteoclast formation. The normal functioning of the epiphyseal growth plate is an important clinical aspect. Much of the physiology of the epiphyseal growth plate in response to exercise includes the important mechanical component. Growth hormone, insulin-like growth factor I, glucocorticoid, thyroid hormone, estrogen, androgen, vitamin D, and leptin are seen as key physiological factors. While there is a need for children to participate in physical activity, clinical consideration needs to be given to how the epiphyseal growth plate functions.

**Conclusions:**

Mechanical loading of the bone is important for epiphyseal plate physiology. Exercise has a healthy function on the normal growth of this important biomechanical feature. Clinically, over-exertion in the form of increased load bearing on the epiphyseal growth plate creates an ideal injury. There is a paucity of research on inactivity on the epiphyseal growth plate resulting in stress deprivation. Further research should take into consideration what lack of exercise and lessened mechanical load bearing has on the function of the epiphyseal growth plate.

**Keywords:**

Child; Physical activity; Epiphyseal growth plates; Bone; Exercise; Mechanical loading

## Introduction

Bone has been described as a dynamic and highly interactive complex of many cell and tissue types [1]. The epiphyseal growth plate is made of several key aspects including cartilaginous, bony, and fibrous components, which act together to achieve longitudinal bone growth [2]. The epiphyseal growth plate is a final target organ for longitudinal growth and results from the cellular process of chondrocyte proliferation and differentiation [3]. Jaramillo and Hoffer [4] described the cartilaginous structures at the ends of growing bones as constituting the "growth mechanism". Frost [5] noted that knowledge of epiphyseal growth plate physiology has application for several areas. The first area noted being that such knowledge aids in distinguishing mechanically competent bone from incompetent bone [5]. The second area is that this knowledge enables a person to increase and maintain bone strength during growth [5]. Finally, knowledge that bone strength, taken in its entirety, and bone health are to be understood as completely different from each other with regards to physiological responses [5]. Of particular importance to clinical practice is the knowledge that two major contributions to the development of articular cartilage are growth factors and mechanical loading [6].

The purpose of this review is to present an overview of the effects of physical activity on the developing epiphyseal growth plates of children. A discussion of the physiological basis of epiphyseal growth plates will be included. Practitioners with a familiarity of the dynamic changes that can occur with the epiphyseal plate in normal children can ultimately lead to recognition of pathologic states [7, 8].

## A Review of the Normal Physiology of the Epiphyseal Growth Plate

As noted previously, skeletal growth at the epiphyseal plate is an active and dynamic process [8]. The epiphyseal growth plate, being a highly specialized layer of cartilage where chondrocytes proliferate and differentiate, brings forth longitudinal bone growth [9]. The epiphyseal growth plate can be divided into three main chondrocyte subpopulations: the resting, proliferating and hypertrophic chondrocytes [10].

Longitudinal bone growth is primarily achieved through the action of chondrocytes in the proliferative and hypertrophic zones of the growth plate [11]. Longitudinal bone growth occurs in the epiphyseal growth plate through a process called endochondral bone formation and ossification [12-14]. Endochondral bone formation is a process where resting zone chondrocytes are recruited to start active proliferation and then undergo differentiation, followed by mineralization [12]. Production of metaphyseal cancellous bone and growth in length are both linked to endochondral ossification with growth plate cartilage cell proliferation as the driving force [15].

Within the epiphyseal growth plate, chondrocyte proliferation, hypertrophy, and cartilage matrix secretion result in chondrogenesis [14]. Endochondral bone development leading at the epiphyseal growth plate contributes to longitudinal bone growth through a process through which undifferentiated mesenchymal cells differentiate into chondrocytes, which then undergo well-ordered and controlled phases of proliferation, hypertrophic differentiation, death, blood vessel invasion, and finally replacement of cartilage with bone [16]. The newly formed cartilage is invaded by blood vessels and bone cells that remodel the newly formed cartilage into bone tissue [14].

The regulation of linear bone growth in children and adolescents comprises a complex interaction of hormones and growth factors [17]. This process of longitudinal bone growth is governed by an intricate network of endocrine signals, including growth hormone, insulin-like growth factor I, glucocorticoid, thyroid hormone, estrogen, androgen, vitamin D, and leptin [14]. Many of these signals act locally on growth plate chondrocytes to regulate epiphyseal growth plate function [14]. The regulation of longitudinal growth at the epiphyseal growth plate occurs generally through the intimate interaction of circulating systemic hormones and locally produced peptide growth factors which has the net result of triggering changes in gene expression by growth plate chondrocytes [13].

In particular, for the majority of skeletal elements to develop and grow, the process of endochondral ossification requires a constantly moving interface between cartilage, invading blood vessels, and bone [1]. An adequate supply of calcium is critical for normal epiphyseal growth plate development and that changes in extracellular calcium modulate the function of chondrocytes with high calcium [Ca2+] leading to cell differentiation [18]. The active metabolite of vitamin D, 1,25-dihydroxyvitamin D (1,25(OH)2D), is classically appreciated to exert its calcemic and other actions via interaction with the vitamin D receptor thus modulating gene transcription [19]. However, with respect to parathyroid gland function and development of the cartilaginous epiphyseal growth plate, calcium and 1,25(OH)2D act cooperatively and 1,25(OH)2D will act independently of the vitamin D receptor [19].

The balance between proliferation and differentiation in bone is considered to be a crucial step. It is a crucial regulatory step controlled by various growth hormones acting in the endocrine pathways [12]. Growth hormone (GH) and insulin-like growth factor-I have major effects on the chondrocytes of the growth plate and act upon all bone cells [20]. Growth hormone (GH), mostly seen in action during the growth spurt in early adolescence, is considered to be the key hormone regulator of linear growth during childhood [17].

Research with laboratory animals has provided most of the current information regarding estrogen's influence on the growth process of long bones, on the maintenance of cancellous bone mass, and on the architectural and cellular changes in bone [21]. Estrogen action is indispensable for normal pubertal growth and growth plate fusion. Both estrogen receptors (ER), ER-alpha and ER-beta, are expressed in the growth plate in boys and girls throughout pubertal development [12]. The rise in estrogen levels at menarche in girls is associated with a large reduction in bone turnover markers and reflects the closure of the epiphyseal growth plates, the reduction in periosteal apposition and endosteal resorption within cortical bone [22]. Jarvinen [23] noted that estrogen tends to pack mechanically-excess mineral into the female skeleton at puberty thus creating the paradigm that the most responsive period of female bone to mechanical loading occurs prior to menarche. This knowledge has consequence for the encouragement of physical activity especially for the female population.

As previously noted, longitudinal growth of the skeleton is a result of endochondral ossification that occurs at the epiphyseal growth plate. Through the sequential process of cell proliferation, extracellular matrix synthesis, cellular hypertrophy, matrix mineralization, vascular invasion, and eventually apoptosis, cartilage continually is being replaced by bone as length increases [15]. Parfitt [15] explained that genetic determination of bone mass is mediated by two classes of gene. The first class of gene (under the control of the sizostat) regulates growth of muscles and bones [15]. The second class of gene (under the control of the mechanostat) regulates the increase in bone density in response to load bearing [15]. With skeletal maturity, there is a decreased growth rate and is mainly associated with structural changes in the physis, including a gradual decline in growth plate width due to the reduced height of the proliferative and hypertrophic zones [13].

## Mechanical Influence on the Physiology of the Epiphyseal Growth Plate

Comprehension of the biomechanical aspects of bone allows one to conceptualize the physiological processes associated with exercise and physical activity on the epiphyseal growth plate. The mechanical influence on bone directly applies to the normal physiological functioning of bone. Longitudinal growth is controlled by local mechanical factors in the form of a feedback mechanism which exists to ensure that bone growth proceeds in the direction of the predominant mechanical forces [11]. The individual epiphyses undergoes a characteristic series of events; central calcification, absorption of cartilage and endochondral ossification, the further course of which is definitely determined by the degree of local distortion [24]. Production of diaphyseal cortical bone and growth in width are both linked to periosteal apposition driven by the process of osteoblast precursor proliferation [15]. During adolescence the trabeculae and cortices become thicker by endosteal apposition which increases bone density [15]. Intrinsically, biophysical forces placed upon the bone assist to develop the growing bone while extrinsic biophysical forces tend to resist and channel the expansion of bone into its functional forms such as the internal trabecular architecture and the external shape [25].

In addition to the vital function of growth factors, it is known that mechanical forces stimulate the synthesis of extracellular proteins in vitro and in vivo and can affect the tissue's overall structure [7]. According to the mechanostat theory, periosteal apposition is regulated by biomechanical requirements [11]. The stress acting on the cartilaginous epiphysis is comparable to that in the adult with relative differences attributed to variations in the mechanical relationship and to the hormonal control of the body's growth [24]. Frost [5] noted that later-discovered tissue-level mechanisms and functions (including biomechanical and muscle) are the true key players in bone physiology, and homeostasis ranks below the mechanical functions.

## The Role of Exercise and Physical Activity on the Epiphyseal Growth Plate

Over the past decade, there has been a surge in the number of sports opportunities available to young athletes [26]. Beyond the positive physiological, psychological and social aspects that a sports activity brings to adolescents, there exists the potential risk of injuries and overuse of the locomotor system [27]. Nonetheless, pursuing sports as a leisure time activity has not been found to be harmful for children [28]. However, this must be coupled with proper instruction, appropriate amounts of physical activity as well as safety.

The effects of exercise on the molecular nature of secreted human growth hormone (GH) or its biological activity are not well understood [29]. Yet it is known that children have more elastic soft tissue and more potential for remodeling than adults [30]. Epiphyseal growth plates are often less resistant to deforming forces than ligaments or joint structures. A child's skeletal system shows pronounced adaptive changes to intensive sports training [31, 32]. The growing skeleton is said to be more responsive than the mature skeleton to the osteotrophic effect of exercise [14]. The mechanstat, a process which is mediated by the detection of deviations from a target value for strain and is a conglomeration of cellular responses that tend to restore target values [15]. From this, the genetic influence on bone mass and density are largely mediated by body size, bone size, and muscle mass [15]. Most long bones end near the joint in a separate epiphysis which at first consists of cartilage and is later ossified [24]. This epiphysis becomes fused with the shaft of the bone and in most cases only at the end of puberty [24]. Thus the stage of growth and development of the child is suggestive of the amount and intensity of exercise that can be performed and tolerated. The recommendation that children, especially adolescents should not perform activities such as plyometrics or engage in heavy weight lifting and should concentrate mainly on such activities that encourages high repetitions at a very low weight [33].

Vascularization of the epiphyseal growth plate region represents a key mechanism for the coupling of two fundamental processes determining the rate of bone growth: chondrogenesis (cartilage production) and osteogenesis (bone formation) [34]. Within the epiphyseal cartilages of such anatomical entities such as the capitellum, trochlea, and medial and lateral epicondyles, vascularity is centripetal [35]. Due to the vascularity pattern it is difficult for avascular necrosis to develop after trauma within the epiphyses [35]. This type of vascularity would represent intraosseous vascularity whereas blood supply outside the epiphyseal plate would represent extraosseous vascularity. This is due in part that there is no true blood supply to the physis but rather the blood supply advances from the blood vessels of the epiphysis and metaphysis and from the perichondrial ring and vessels of the periosteum [36].

## Influence of Over-activity on the Epiphyseal Growth Plate

A potential problem with physical activity and exercise on the epiphyseal plates is over-activity. Intuitively, it is the extent that over-physical activity may have on the growth plate resulting in injury. A better appreciation of how epiphyseal plate physiology works is seen in the response to trauma. Approximately 25% of adolescents have at least one recreational injury which is mostly minor reflecting only soft tissue trauma and abrasions of the skin [37]. However, approximately 15% of children with fractures involve physeal injuries with 10% of these physeal injuries being sport-related [36]. The most prevalence of epiphyseal growth plate injuries is to children ages 10 to 16 years [38, 39]. If injury occurs to the epiphyseal growth plate the possibility that there may be a premature locking of the epiphyseal growth plate essentially halting bone lengthening [40].

Most injuries in children's sports and activities are minor and self-limiting thus suggestive that children and youth sports are safe [32]. Unlike adults, many of the injuries may be treated closed due to the growth and remodeling potential of children [41]. Skeletally immature children who participate in extreme levels of sports participation can sustain repetitive trauma [42]. This repetitive trauma can cause the epiphyseal plate to widen. Laor et al [42] hypothesized that the metaphyseal vascular supply is disrupted causing the normal process of endochondral bone formation due to long columns of hypertrophic cartilage cells from the physis extending into the metaphysis As the risk of injuries sustained by young athletes can be significant, it is essential that training programs take into account physical and psychological immaturity, so that the growing athlete can adjust to their own body changes [31]. The period of early puberty is associated with an increased risk of fracture which may be related to the high rate of bone turnover [22]. A late menarche is a consistent risk factor for fracture in young females due to hormonal instability that may affect bone density [22]. Growth disturbance depends on extent of the injury and the amount of remaining growth potential [36]. However, it can be hypothesized that a partially closed physis are weak links in children and that asymmetrically or partially closed physis may be vulnerable to trauma [43]. It has been suggested that overuse during puberty for females may result in long term development of low bone density and ongoing problems with bone health.

The genetic potential for bone accumulation can be frustrated by insufficient calcium intake, disruption of the calendar of puberty and inadequate physical activity [15]. While many of the molecular mechanisms that control cellular differentiation and growth during embryogenesis recur during fracture healing taking place in a post-natal environment that is unique and distinct from those which exist during embryogenesis [44]. Disruption of either the longitudinal intraosseous vasculature (vertical extraosseous blood supply) or the vascular arch in more than two places may lead to selective avascular necrosis (extraosseous) of the epiphyseal cartilage [35].

The clinical pathophysiology of excessive activity on the epiphyseal growth plates resulting in injury is one of the more prominent methods of understanding the effects of exercise on growth plate physiology. The immature skeleton is different from the adult skeleton with unique vulnerability to acute and chronic injuries at the growth plate [7]. Epiphyseal injuries are usually due to shearing and avulsion forces as well as compressive forces usually due to either severe twisting or direct blows that can result in a disruption of the epiphyseal growth plate [31]. In young athletes, as the bone stiffness increases and resistance to impact diminishes, sudden overload may subject bone to either bow or buckle [31]. Swelling, hyperemia, and deformity in the physeal area are the classical signs of physeal injury with pain being potentially less intense [36]. Physeal injuries occur in 15% of children with fractures, and 10% of all physeal injuries are sport-related [36].

Gerstenfeld et al [44] summarized five key points of damaged epiphyseal plate healing that needs to be considered clinically. First, the anatomic structure of callus formation as it progresses during the healing phases should be considered [44]. Second, morphogenetic signals that facilitate the repair process should be known [44]. Third, and of importance for clinicians, is the role of the biomechanical aspect in controlling differentiation during cellular repair [44]. Fourth, the role of key groups of soluble factors i.e. pro-inflammatory cytokines and angiogenic factors during repair are important for establishing vascularity [44]. Finally, knowledge and appreciation for the relationship between the genetic components that control bone mass and remodeling is warranted [44]. These five key points should be acknowledged clinically in an effort to monitor for proper healing post-injury to the epiphyseal plate. From these key points, one can gain an appreciation for not reducing sport activity but in the intensity of the sport activity during the high intense growth phases. For example, it is not uncommon to see adolescents training year-round in one specific sport without the ability to either rest during the off-season or enter another sport that may be different and/or less intense in the training. As well, prohibitions of negative training and in some cases, the prohibition of sports all together, are sometimes necessary to minimize the potential for injury [27]. While sports are important for children, safety and prevention of needless injury should be considered.

## Influence of Inactivity on the Epiphyseal Growth Plate

The second and arguably the least discussed aspect concerning the epiphyseal growth plate is the role inactivity may play on the growth plate. Given the current interest in and rising rates of child obesity such interest in the growth plate should be considered. One of the implicated culprits in the child obesity epidemic is the lack of physical activity. It is known that epiphyseal growth plate activity controls longitudinal bone growth and leads ultimately to adult height yet numerous disorders are characterized by retarded growth and reduced final height either have their origin in altered chondrocyte physiology or display pathological growth plate changes secondary to other causes [45]. Physical activity may have a protective effect on the epiphyseal growth plate, however, very little research has been conducted on the role of inactivity on the epiphyseal growth plates. This is possibly due to the lack of clinically-related biomechanical problems that emanate from a lack of physical activity on the epiphyseal plates. In other words, there is a paucity of research in the form of case studies that has implicated a lack of physical activity as an etiology for epiphyseal growth plate injury. Despite this, one could hypothesize that physical inactivity would not serve as an effect mechanism of protection. Frost [5] observed this very phenomenon but noted that for an obese person the stronger muscles would put larger loads on bones to which bone physiology should respond by increasing bone strength even if non-mechanical factors are involved. Until further research is undertaken on the effects of inactivity on the growth plate, one can extrapolate such effects from the current literature on normal and excessive functioning that sedentarism may result in inadequate stimulation of the growth plate with a possible result of changed growth potential.

Nonetheless, the recent concern over the increasing incidence and prevalence of obesity as seen in children gives rise to concern for the normal growth process. As previously indicated, functioning growth hormone (GH) and insulin-like growth factor (IGF)-I are essential for normal growth [46]. However, obese children will typically grow at a normal rate despite the presence of low serum GH levels with leptin, insulin, and sex hormones working to locally activate the IGF system at the epiphyseal plate [46]. Serum leptin may play a biological role in regulating bone metabolism by increasing the proliferation and differentiation of osteoblasts in adults [47]. Phillip et al [46] found that an elevated level of leptin in obese children can affect the bone growth center and it may be that leptin also participates in growth without GH observed in obesity.

It may appear that, in some cases, genetic expression, through favorable conditions, can be maximally achieved throughout the entire period of growth [48]. In this instance, it is hypothesized that for harm to be placed on the growth plate or show delayed growth maturation at the epiphyseal growth plate for those children who are physically inactive, the combination of genetic expression through unfavorable environmental and socioeconomic conditions may be the culprit in abnormal epiphyseal growth plate physiology. In other words, only through unfavorable conditions such as extreme poverty, lack of nutrition, and other entities will the genetic expression manifest itself for those children who are physically inactive. While it is known that load-bearing tissue, such as articular cartilage, will atrophy in the absence of mechanical forces [6], future investigation into the effects of a lack of load bearing through a lack of physical activity on the epiphyseal plate is warranted.

## Conclusion

The epiphyseal growth plate is a dynamic entity. Growth is dependent not only on intrinsic factors such as hormones and other regulatory factors but on extrinsic factors. These extrinsic factors are based entirely on the biomechanical model. Exercise, a positive aspect for the epiphyseal growth plate needs to be moderated through carefully crafted activities especially during pubertal growth spurts. Obesity, a major problem among today's youth, can be attributed in part to a lack of exercise. Once activity is undertaken the potential for epiphyseal growth plate disturbances from too much activity may be a predisposing factor to growth plate dynamics. The effects of exercise on the epiphyseal growth plate needs further research to comprehend the entirety of this dynamic anatomical and physiological entity. Research in the area of the epiphyseal growth plate in some children who are sedentary needs to be addressed.
